# Differential Intestinal Mucosa Transcriptomic Biomarkers for Crohn's Disease and Ulcerative Colitis

**DOI:** 10.1155/2018/9208274

**Published:** 2018-10-17

**Authors:** Maria Dobre, Elena Milanesi, Teodora Ecaterina Mănuc, Dorel Eugen Arsene, Cristian George Ţieranu, Carlo Maj, Gabriel Becheanu, Mircea Mănuc

**Affiliations:** ^1^Victor Babes National Institute of Pathology, 050096 Bucharest, Romania; ^2^Fundeni Clinical Institute, 022328 Bucharest, Romania; ^3^National Institute of Neurology and Neurovascular Diseases, 041914 Bucharest, Romania; ^4^Carol Davila University of Medicine and Pharmacy, 050474 Bucharest, Romania; ^5^Elias Emergency University Hospital, 011461 Bucharest, Romania; ^6^Institute for Genomic Statistics and Bioinformatics, University Hospital Bonn, Germany

## Abstract

Genetic research has shaped the inflammatory bowel disease (IBD) landscape identifying nearly two hundred risk loci. Nonetheless, the identified variants rendered only a partial success in providing criteria for the differential diagnosis between ulcerative colitis (UC) and Crohn's disease (CD). Transcript levels from affected intestinal mucosa may serve as tentative biomarkers for improving classification and diagnosis of IBD. The aim of our study was to identify gene expression profiles specific for UC and CD, in endoscopically affected and normal intestinal colonic mucosa from IBD patients. We evaluated a panel of 84 genes related to the IBD-inflammatory pathway on 21 UC and 22 CD paired inflamed and not inflamed mucosa and on age-matched normal mucosa from 21 non-IBD controls. Two genes in UC (CCL11 and MMP10) and two in CD (C4BPB and IL1RN) showed an upregulation trend in both noninflamed and inflamed mucosa compared to controls. Our results suggest that the transcript levels of CCL11, MMP10, C4BPB, and IL1RN are candidate biomarkers that could help in clinical practice for the differential diagnosis between UC and CD and could guide new research on future therapeutic targets.

## 1. Introduction

Inflammatory bowel diseases (IBD) are a distinct class of gastrointestinal diseases mainly represented by Crohn's disease (CD) and ulcerative colitis (UC). These are chronic diseases characterized by a relapsing remitting course with an increasingly high incidence and prevalence worldwide [1]. The current accepted model for IBD etiology implies the existence of a genetic predisposition, perturbations in the intestinal barrier components, and altered microbiota, which combined will lead to an aberrant immune response [2]. Distinguishing between the two diseases represents a problem in clinical practice due to some similarities in endoscopic and morphological aspects which in turn will lead to a change in diagnosis throughout the course of disease [3]. However, some fundamental differences between CD and UC have been reported: UC is characterized by diffuse inflammation confined to the colorectal mucosa, whereas in CD, the inflammation is discontinuous, transmural, and can affect the entire gastrointestinal tract. Moreover, CD patients often present complications like intestinal strictures, fistulas, and abscesses [4]. Despite these differences, physiopathological mechanisms, clinical criteria, and therapeutical strategies considerably overlap, but CD and UC seem to be triggered and maintained by differential molecular mechanisms, which are not completely known.

Genetic studies in IBD have gained importance during the past decade since endoscopic assessment and biopsies provide limited data regarding early disease activity and factors for relapse. The candidate gene approach, genome-wide association studies, and meta-analyses have contoured the genetic background of these disorders, revealing more than 200 risk loci in both European and non-European individuals [5]. However, previous studies showed that many of these loci are shared between CD and UC [6], and no specific genetic markers entered clinical practice yet.

A number of candidate gene expression studies, RNA sequencing, and microarray studies on mucosa from IBD patients have been published in the last years with the attempt to find a specific profile able to discriminate UC and CD. Gene expression analysis of tissue samples from affected and nonaffected individuals can help in discovering important events involved in disease pathogenesis. For example, individual mRNA levels can be sensitive markers for improving classification and diagnosis, identifying new therapeutic targets, and providing prognostic information [7].

Studies conducted so far analyzing the expression levels of cytokines and transcription factors in mucosa revealed that CD has been associated with an impairment of Th1/Th17 response [8], whereas UC has been associated with a Th2/NKT cell response [9]. Other genes have been indicated as putative differential biomarkers, including *α*-defensin-5 [10], circadian genes [11], TNFAIP3, PIGR, TNF, and PIGR [12]. Other studies based on RNA-seq approaches revealed important transcriptomic differences between normal mucosa, noninflamed CD mucosa, and inflamed CD mucosa [13] as well as differences among colon biopsies from CD patients, UC patients, and non-IBD controls [4].

In this study, we aimed to identify the inflammatory signature specific for UC and CD both in endoscopically inflamed and not inflamed mucosa and how the type of therapy can influence the gene expression profile in Romanian patients. To address these questions, we evaluated the gene expression profile of a panel of 84 selected genes (previously associated to IBD) in paired mucosa samples of 21 UC and 22 CD patients, and we compared them with the profiles obtained in a group of 21 non-IBD healthy controls.

## 2. Materials and Methods

### 2.1. Patients

Forty-three IBD patients (21 UC and 22 CD) and 21 non-IBD controls have been enrolled in the study at the Department of Gastroenterology and Hepatology, “Elias” Emergency University Hospital and at the “Fundeni” Clinical Institute of Bucharest, Romania. In terms of disease location, patients with CD had colonic and ileocolic forms of the disease. All the patients and controls were of Romanian origin. Written informed consent was obtained from all participants prior to biopsy collection, and the study was approved by the local ethics committees. The diagnosis had been made based on clinical, endoscopic, and histological criteria according to European Crohn's and Colitis Organization Guidelines [3]. From each UC and CD patients, paired colonic inflamed mucosa (IM) and macroscopically colonic noninflamed mucosa (NM) were obtained during a colonoscopy. We defined the inflammation status based on the presence of erythema, ulcerations, and bleeding of the mucosa. A biopsy of a normal-looking colonic mucosa was obtained also from a group of non-IBD controls during a colonoscopy screening. Exclusion criteria for non-IBD controls were as follow: (1) presence of digestive symptoms, (2) current or previous nonsteroidal anti-inflammatory treatments (within the past 3 months), and (3) current or previous anticoagulant/antiplatelet treatments (within the past 3 months). The characteristics of the three groups are reported in Table 1.

### 2.2. Total RNA Isolation and qPCR

Total RNA isolation from fresh-frozen tissues preserved in RNA later was performed using RNeasy mini Kit (Qiagen), according to the manufacturer's protocols. The quantity and quality of RNA were determined using the NanoDrop 2000 (Thermo Scientific). An amount of 600 ng of RNA was reverse transcribed to cDNA using the RT2 First Strand Kit (Qiagen). The Human Crohn's Disease RT2 Profiler PCR Array (PAHS-169Z, Qiagen), using SYBR Green chemistry, evaluated the expression of 84 key genes, according to the manufacturer's protocol, on the ABI-7500 fast instrument (Applied Biosystems). The expression levels of each gene were normalized on the geometric mean values of two housekeeping genes (GAPDH and HPRT1) based on RefFinder algorithm (http://leonxie.esy.es/RefFinder/) [14] analysis of five candidate reference genes (ACTB, B2M, GAPDH, HPRT1, and RPLP0).

### 2.3. Statistical Analysis

qRT-PCR data analysis was conducted using the Statistical Package for Social Science (SPSS version 17.0). Categorical variables were tested by means of the chi-square test, and continuous variables with the *t*-test. Paired *t*-test was used to assess difference in gene expression levels of IM and NM.

## 3. Results

The group of patients and controls was homogeneous for age (*p* > 0.05) and sex (*χ*^2^ = 4.880, *p* = 0.087) distribution, and the UC and CD groups did not statistically differ for the class of treatment (*χ*^2^ = 6.409, *p* = 0.171).

### 3.1. Gene Expression Alterations in Paired Inflamed and Noninflamed Mucosa of UC and CD Patients

Gene expression analysis was performed on 21 pairs of tissues representing IMUC and NMUC and 22 pairs of tissues representing IMCD and NMCD. In IM, 11 genes out of 84 were found differentially overexpressed both in UC and CD compared with the paired NM. Thirty-three transcripts were found specifically altered only in UC patients (two downregulated and 31 upregulated). Results are shown in Table 2.

### 3.2. Gene Expression Alterations in CD and UC Patients Compared with Non-IBD Controls

**Figure 1:**
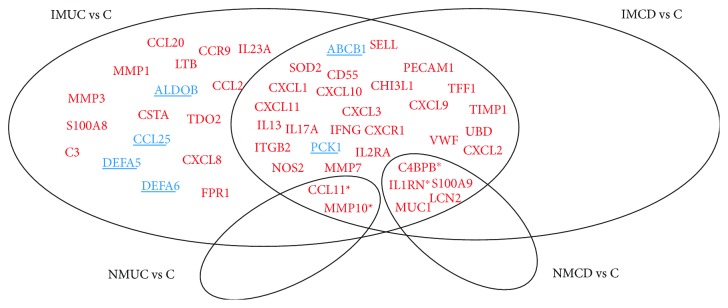
Venn diagram showing the genes differentially expressed across the performed different comparisons with controls. Genes marked with asterisk (^∗^) are differentially expressed in the paired IM-NM analysis. IM = inflamed mucosa; NM = noninflamed mucosa.

**Figure 2:**
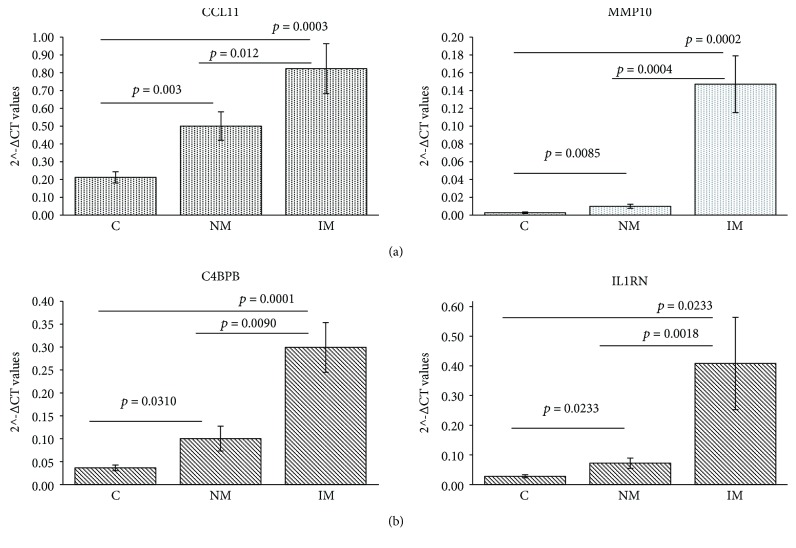
Bar graphs represent the mean of the 2^-ΔCT values, and error bars represent the standard error. The graphs show the genes differentially expressed comparing inflamed mucosa (IM) vs. noninflamed mucosa (NM) IM vs. NM (paired), IM vs. C, and NM vs. C in UC (a) and in CD (b). C = non-IBD controls; IM = inflamed mucosa; NM = noninflamed mucosa.

Gene expression analysis was performed on 21 noninflamed and inflamed mucosa from UC patients, 22 from CD, and 21 from healthy controls. Considering a fold change (FC) > |2.0| and a *p* value below 0.05, 32 genes out of 84 were found differentially expressed both in UC and CD compared with C (two downregulated and 30 upregulated), and 17 were specifically altered only in UC patients (four downregulated and 13 upregulated). No gene was found modified only in CD. When comparing the NM tissues vs. C, we found two transcripts upregulated in UC and five upregulated in CD (Table 3). A graphic representation of the results is shown in Figure 1. Genes whose expression differed between NM and controls and are also different comparing paired IM-NM are shown in Figures 2(a) and 2(b).

### 3.3. Differences in Gene Expression in IBD Patients on Different Treatments

Due to the limited sample size of the UC and CD groups, we analyzed the treatment effect on gene expression levels considering the entire IBD cohort. Comparing the patients treated with 5-ASA (*n* = 21) vs. drug-free patients (*n* = 7), we found that ISG15 ubiquitin-like modifier (ISG15) was downregulated both in inflamed and not inflamed tissues with FC and *p* value of −2.04, *p* = 0.003 in IM and −1.84, *p* = 0.033 in NM. Moreover, we found that the six patients with biologic treatment showed lower levels of serum amyloid A1 (SAA1) with FC of −6.66 and *p* = 0.025 in IM.

Comparing patients with biological treatment vs. 5-ASA, we found that CCR1 was upregulated in IM with FC = 2.1 and *p* = 0.005 and TFF1 was downregulated both in IM and NM with FC = −2.5, *p* = 0.001 and FC = −2.4, *p* = 0.004, respectively.

Despite the limited size of the two groups, an additional analysis to find a putative effect of the treatment on the candidate genes (IL1RN and C4BP4 for UC and CCL11 and MMP10 for CD) has been performed separately both on UC and CD groups. No changes in IL1RN and C4BP4 levels were found between the three UC patients without treatment and the UC patients in treatment with 5-ASA (*p* = 0.704, *p* = 0.718), biological treatment (*p* = 0.384, *p* = 0.567), or polytherapy (*p* = 0.891, *p* = 0.680). In the CD group, no difference in CCL11 and MMP10 was found comparing the four patients without treatment and the other groups (*p* > 0.05 in all the comparisons). However, a trend toward significance was observed in MMP10 levels comparing the 4 CD patients without treatment and the group of the seven patients using 5-ASA (*p* = 0.056).

## 4. Discussion

Overlapping features have been reported in up to 30% of IBD [15] leading to a not accurate diagnosis and increasing the risk of inappropriate treatment. In this study, we sought to determine whether mucosal gene profile could be used to develop diagnostic biomarker(s) to discriminate between the two main inflammatory bowel diseases (UC and CD) more accurately.

To the best of our knowledge, this is the first study that evaluated 84 transcripts by qRT-PCR considering a larger cohort of participants than previous studies, including paired inflamed and not inflamed tissues from CD and UC as well as a cohort of non-IBD controls.

Using this approach, we identified 17 genes differentially expressed only in the inflamed mucosa from UC that did not differ for the CD patients. A common signature of 32 genes was identified, and no gene specific for CD inflamed mucosa was found.

Among the genes belonging to the common signature, five and two were found differentially expressed comparing the not inflamed mucosa with mucosa from non-IBD controls of CD and UC, respectively.

Interestingly, in UC, CCL11 and MMP10 were increased substantially in non-IBD controls, NM and IM, whereas in CD, this increase was observed for C4BPB and IL1RN. Hence, these four genes seem to be specific markers of UC and CD inflammation levels.

Eotaxin-1 (CCL11), a potent eosinophil chemoattractant that is considered a major contributor to tissue eosinophilia, is a key regulator of intestinal inflammation [16] and seems to be involved both in UC and CD. Indeed, unlike other chemokines, the human mRNA for eotaxin-1 is constitutively expressed in the small intestine and colon [17] where the intestinal myeloid cells seem to be a source [18].

Levels of eotaxin-1 have been found increased in sera from UC patients [19–21] as well as in colon biopsies [22]. In line with our findings that suggested an increase according to the inflammation status, a significant increase of its levels was found in patients with active UC but not in the quiescent state [23]. These data suggest that also the peripheral levels may increase accordingly to the inflammation grade as we observed in mucosa.

Increased levels of eotaxin-1 have been found also in the sera from CD patients [19, 20], and our group found that its mucosal mRNA levels were higher in active CD than in controls. However, no changes were observed in the remission state [24] or in UC [25].

Another transcript having a similar trend like CCL11 in UC was MMP10. MMP10 belongs to the human matrix metalloproteinases family consisting of 24 zinc-dependent endopeptidases and is produced by infiltrating myeloid cells. Their levels are transcriptionally upregulated in response to proinflammatory cytokines, and both transcripts and protein levels of some MMPs are demonstrated to be upregulated in inflamed mucosa or serum of IBD patients [26, 27] even in the naïve to treatment subgroup [28]. In addition, increased expression of epithelial MMP10 has been found in colonic mucosa of both UC and CD pediatric patients compared to non-IBD patients [29]. MMP10 was seen as a possible therapeutic target in IBD because its expression had been observed close to the edges of healing ulcers in human specimens of UC [30]. Its influence, however, can be debated since it could have a role in disease resolution but also in the proinflammatory process. In animal models of experimental colitis, MMP10 seems to promote mucosal healing, and in its absence due to persistent colonic inflammation, dysplastic lesions could be promoted [31]. Human genetic studies identified six SNPs across the MMP10 gene associated with UC, suggesting that these genetic variants may play a role in UC susceptibility and clinical outcome [32].

Moving forward to the specific genes associated to CD in our cohort, C4BPB and IL1RN, they will be discussed below.

The C4BPB gene encodes for C4b-binding protein, a multimeric protein that controls the complement cascade. There is one single study for this gene in CD which evaluated the serum level of C4BPB in patients treated with infliximab, revealing that upregulation of this protein is associated with primary nonresponse to this treatment [33]. Our investigation took into account current biologic treatment, but none of the patients included had had a nonresponse status declared. Thus, we can only suggest that increased expression can only be attributed to the inflammatory process.

Finally, our analysis associated the IL1RN (interleukin 1 receptor antagonist) gene with inflammation in CD. The IL1RN gene encodes for a protein member of the interleukin 1 cytokine family. This protein inhibits the interleukin 1 alpha and beta activities and modulates a variety of related immunoinflammatory responses.

Discordant results regarding the associations between IL1RN genetic variants and IBD have been published. Some studies reported significant associations with CD [34, 35] and UC predispositions [36, 37], treatment outcome [38], and age at the onset [39]; on the contrary, other studies did not find any associations [40–42]. Interestingly, IL-1RN^∗^2 variant has been associated with reduced levels of IL-1ra protein and IL-1RN mRNA in the colonic mucosa from UC patients [43].

Summarizing, against our expectations, only four putative candidate biomarkers able to discriminate UC and CD were found. This can be due to the large gene expression intravariability observed both in the colonic mucosa from non-IBD and IBD groups. Indeed, due to a number of parameters not yet included (histologically active/in remission, duration, and response to treatment), this group intravariability might have increased. Furthermore, the raw data reported that a larger number of genes seemed to be differentially expressed (with high fold difference) without reaching statistical significance due to the high standard deviation. Accordingly, in order to find a more specific signature, the study should be validated in a larger, more homogenous cohort.

Another aim of this study was to evaluate the influence of treatment on the entire IBD cohort. Our results showed a downregulation of ISG15 in patients treated with 5-ASA and a downregulation of SAA 1 in patients with biologic treatment compared to patients without IBD treatment. The effect of different therapeutic agents on IBD gene expression should be assessed in a longitudinal cohort.

The main limitation of this study was the absence of data regarding the clinical scores (MAYO and CDAI) measuring the activity and severity of IBD.

## 5. Conclusions

In conclusion, we obtained differential intestinal mucosa expression signatures of 17 genes that could specifically characterize the UC inflamed mucosa. Of note, two genes in UC (CCL11 and MMP10) and two in CD (C4BPB and IL1RN) had significantly upregulated expression in the noninflamed and inflamed mucosa compared to controls. Our putative biomarkers, once validated in a larger cohort, could help in clinical practice for the differential diagnosis between UC and CD and could guide new researches on future therapeutic targets.

## Figures and Tables

**Table 1:** Clinical and demographical parameters of individuals involved in the study.

	CTRL (*n* = 21)	UC (*n* = 21)	CD (*n* = 22)
*Sex, n (%)*			
Male	9 (42.9)	16 (76.2)	13 (59.1)
Female	12 (57.1)	5 (23.8)	9 (40.9)
*Age, yrs, mean ± SD*	46.5 ± 16.7	44.4 ± 12.8	45.1 ± 15.1
*Medications at tissue acquisition n (%)*			
None	21 (100)	3 (14.3)	4 (18.2)
Biological	—	2 (9.5)	4 (18.2)
5-ASA	—	14 (66.7)	7 (31.8)
Cortisone	—	—	2 (9.1)
Polytherapy	—	2 (9.5)	5 (22.7)

**Table 2:** The table shows the transcripts that differed by >2.0 fold with *p* < 0.05 in inflamed mucosa (IM) vs. noninflamed mucosa (NM) in UC and CD patients. Genes are arranged by alphabetic order. Italic fonts indicate genes differentially expressed only in IM from UC.

Gene	Description	UC		CD	
		Paired IM vs. NM		Paired IM vs. NM	
		FC	*p* value	FC	*p* value
*C3*	Complement C3	5.48	0.0106		
C4BPB	Complement component 4 binding protein beta	5.86	<0.0001	2.98	0.0090
CCL11	C-C motif chemokine ligand 11	2.01	0.0121	2.27	0.0045
*CCL20*	C-C motif chemokine ligand 20	4.05	0.0003		
*CD55*	CD55 molecule (Cromer blood group)	3.63	<0.0001		
CHI3L1	Chitinase 3 like 1	16.96	0.0045	4.19	0.0328
*CR2*	Complement C3d receptor 2	7.00	0.0150		
CXCL1	C-X-C motif chemokine ligand 1	17.99	<0.0001	6.82	0.0413
*CXCL10*	C-X-C motif chemokine ligand 10	3.23	0.0008		
CXCL11	C-X-C motif chemokine ligand 11	8.93	0.0001	4.32	0.0380
*CXCL2*	C-X-C motif chemokine ligand 2	14.98	<0.0001		
*CXCL3*	C-X-C motif chemokine ligand 3	9.79	<0.0001		
CXCL9	C-X-C motif chemokine ligand 9	5.16	0.0002	5.22	0.0059
*CXCR1*	C-X-C motif chemokine receptor 1	19.22	0.0131		
*EDN3*	Endothelin 3	−2.92	0.0048		
*FPR1*	Formyl peptide receptor 1	10.57	0.0035		
*IFNG*	Interferon gamma	2.82	<0.0001		
IL1RN	Interleukin 1 receptor antagonist	8.15	0.0018	5.68	0.0498
*IL23A*	Interleukin 23 subunit alpha	3.11	<0.0001		
*IL2RA*	Interleukin 2 receptor subunit alpha	3.45	0.0006		
*CXCL8*	C-X-C motif chemokine ligand 8	19.40	0.0185		
*ITGB2*	Integrin subunit beta 2	2.31	0.0003		
*LCN2*	Lipocalin 2	13.05	0.0003		
*LTB*	Lymphotoxin beta	4.01	0.0007		
LYZ	Lysozyme	2.06	0.0009	2.16	0.0344
*MMP1*	Matrix metallopeptidase 1	9.98	0.0108		
*MMP10*	Matrix metallopeptidase 10	14.92	0.0004		
*MMP3*	Matrix metallopeptidase 3	30.01	0.0016		
MMP7	Matrix metallopeptidase 7	37.37	0.0036	6.00	0.0098
*NOS2*	Nitric oxide synthase 2	10.99	0.0005		
*PCK1*	Phosphoenolpyruvate carboxykinase 1	−6.29	0.0002		
*PECAM1*	Platelet endothelial cell adhesion molecule 1	2.42	0.0046		
*REG1A*	Regenerating family member 1 alpha	10.11	0.0123		
*S100A8*	S100 calcium binding protein A8	17.91	0.0018		
*S100A9*	S100 calcium binding protein A9	9.31	0.0005		
*SAA1*	Serum amyloid A1	62.83	0.0016		
*SELL*	Selectin L	5.31	<0.0001		
SOD2	Superoxide dismutase 2	2.14	0.0003	2.03	0.0429
*STAT1*	Signal transducer and activator of transcription 1	2.26	<0.0001		
*TDO2*	Tryptophan 2,3-dioxygenase	4.00	<0.0001		
*TFF1*	Trefoil factor 1	2.74	0.0001		
*TIMP1*	TIMP metallopeptidase inhibitor 1	4.48	<0.0001		
*TNF*	Tumor necrosis factor	2.58	0.0022		
UBD	Ubiquitin D	7.95	0.0002	2.92	0.0116

**Table 3:** The table shows the transcripts that differed by >2.0 fold with *p* < 0.05 in inflamed mucosa (IM) and noninflamed mucosa (NM) of UC and CD patients compared with healthy controls. Genes are arranged by alphabetic order. Italic fonts indicate genes differentially expressed both in NM and IM compared to controls.

Gene	Description	UC				CD			
		IM (*n* = 21) vs. C (*n* = 21)		NM (*n* = 21) vs. C (*n* = 21)		IM (*n* = 22) vs. C (*n* = 21)		NM (*n* = 22) vs. C (*n* = 21)	
		FC	*p* value	FC	*p* value	FC	*p* value	FC	*p* value
ABCB1	ATP binding cassette subfamily B member 1	−7.04	0.0158			−3.76	0.0417		
ALDOB	Aldolase, fructose-bisphosphate B	−18.72	0.0269						
C3	Complement C3	3.77	0.0264						
C4BPB	Complement component 4 binding protein beta	10.05	<0.0001			8.21	<0.0001	2.76	0.0310
*CCL11*	C-C motif chemokine ligand 11	3.99	0.0003	2.056	0.003	3.90	<0.0001		
CCL2	C-C motif chemokine ligand 2	2.58	0.0413						
CCL20	C-C motif chemokine ligand 20	3.36	0.0009						
CCL25	C-C motif chemokine ligand 25	−13.47	0.0403						
CCR9	C-C motif chemokine receptor 9	−2.89	0.0179						
CD55	CD55 molecule (Cromer blood group)	4.63	<0.0001			2.80	0.0006		
CHI3L1	Chitinase 3 like 1	42.55	0.0016			39.26	0.0049		
CSTA	Cystatin A	2.64	0.0045						
CXCL1	C-X-C motif chemokine ligand 1	12.34	<0.0001			12.60	0.0240		
CXCL10	C-X-C motif chemokine ligand 10	2.85	0.0013			9.36	0.0416		
CXCL11	C-X-C motif chemokine ligand 11	7.28	0.0003			12.59	0.0077		
CXCL2	C-X-C motif chemokine ligand 2	9.16	<0.0001			10.09	0.0368		
CXCL3	C-X-C motif chemokine ligand 3	7.17	<0.0001			5.74	0.0123		
CXCL9	C-X-C motif chemokine ligand 9	5.36	<0.0001			11.04	0.0016		
CXCR1	C-X-C motif chemokine receptor 1	50.54	0.0103			53.31	0.0152		
DEFA5	Defensin alpha 5	−11.68	0.0422						
DEFA6	Defensin alpha 6	−12.36	0.0368						
FPR1	Formyl peptide receptor 1	11.09	0.0031						
IFNG	Interferon gamma	2.89	0.0001			3.11	0.0287		
IL13	Interleukin 13	4.08	0.0046			2.82	0.0083		
IL17A	Interleukin 17A	4.31	0.0032			2.39	0.0048		
*IL1RN*	Interleukin 1 receptor antagonist	12.45	0.0011			14.78	0.0233	2.6	0.0233
IL23A	Interleukin 23 subunit alpha	3.26	0.0013						
IL2RA	Interleukin 2 receptor subunit alpha	2.61	0.0026			2.44	0.0073		
CXCL8	C-X-C motif chemokine ligand 8	24.75	0.0169						
ITGB2	Integrin subunit beta 2	2.32	0.0005			1.75	0.0126		
*LCN2*	Lipocalin 2	19.25	0.0002			13.56	0.0006	5.957	0.0074
LTB	Lymphotoxin beta	2.91	0.0061						
MMP1	Matrix metallopeptidase 1	16.79	0.0074						
*MMP10*	Matrix metallopeptidase 10	53.71	0.0002	3.6	0.0085	22.84	<0.0001		
MMP3	Matrix metallopeptidase 3	52.13	0.0013						
MMP7	Matrix metallopeptidase 7	286.33	0.0027			87.38	0.0030		
*MUC1*	Mucin 1, cell surface associated	2.65	<0.0001			2.79	0.0017	2.14	0.0024
NOS2	Nitric oxide synthase 2	11.93	0.0006			7.09	<0.0001		
PCK1	Phosphoenolpyruvate carboxykinase 1	−6.49	<0.0001			−2.36	0.0077		
PECAM1	Platelet and endothelial cell adhesion molecule 1	3.09	0.0021			2.57	0.0060		
S100A8	S100 calcium binding protein A8	28.57	0.0014						
*S100A9*	S100 calcium binding protein A9	16.56	0.0002			33.97	0.0364	3.61	0.0257
SELL	Selectin L	3.87	0.0003			3.97	0.0418		
SOD2	Superoxide dismutase 2	2.17	0.0005			2.33	0.0166		
TDO2	Tryptophan 2,3-dioxygenase	3.88	<0.0001						
TFF1	Trefoil factor 1	3.06	0.0001			2.91	0.0440		
TIMP1	TIMP metallopeptidase inhibitor 1	6.12	<0.0001			4.21	0.0011		
UBD	Ubiquitin D	5.19	0.0006			4.99	0.0007		
VWF	Von Willebrand factor	3.17	<0.0001			2.84	0.0022		

## Data Availability

The data used to support the findings of this study are included within the article.
